# Design and Development of a Novel Upper-Limb Cycling Prosthesis

**DOI:** 10.3390/bioengineering4040089

**Published:** 2017-11-16

**Authors:** Akira Tiele, Shivam Soni-Sadar, Jack Rowbottom, Shilen Patel, Edward Mathewson, Samuel Pearson, David Hutchins, John Head, Stephen Hutchins

**Affiliations:** 1 School of Engineering, University of Warwick, Coventry CV4 7AL, UK; Shivam.Soni@warwick.ac.uk (S.S.-S.); J.Rowbottom@warwick.ac.uk (J.R.); Shilen.Patel@warwick.ac.uk (S.P.); E.Mathewson@warwick.ac.uk (E.M.); Samuel.Pearson@warwick.ac.uk (S.P.); D.A.Hutchins@warwick.ac.uk (D.H.); 2 School of Health Sciences, University of Salford, Salford M5 4WT, UK; J.Head@salford.ac.uk; 3 Department of Occupational Therapy, Prosthetics and Orthotics, Oslo and Akershus University College of Applied Sciences, NO-0130 Oslo, Norway; Stephen.Hutchins@hioa.no

**Keywords:** sports prosthesis, upper-limb amputees, terminal device, cycling, rehabilitation

## Abstract

The rise in popularity of the Paralympics in recent years has created a need for effective, low-cost sports-prosthetic devices for upper-limb amputees. There are various opportunities for lower-limb amputees to participate in cycling; however, there are only few options for those with upper-limb amputations. If the individual previously participated in cycling, a cycling-specific prosthesis could allow these activities to be integrated into rehabilitation methods. This article describes the processes involved with designing, developing and manufacturing such a prosthesis. The fundamental needs of people with upper-limb amputation were assessed and realised in the prototype of a transradial terminal device with two release mechanisms, including a sliding mechanism (for falls and minor collisions) and clamping mechanism (for head-on collisions). The sliding mechanism requires the rider to exert approximately 200 N, while the clamping mechanism requires about 700 N. The force ranges can be customised to match rider requirements. Experiments were conducted in a controlled environment to demonstrate stability of the device during normal cycling. Moreover, a volunteer test-rider was able to successfully activate the release mechanism during a simulated emergency scenario. The development of this prosthesis has the potential to enable traumatic upper-limb amputees to participate in cycling for rehabilitation or recreation.

## 1. Introduction

There is a global public health problem relating to physical inactivity, which has been attributed to approximately 3.2 million annual deaths [1]. In 2012, only 41% of men and 31% of women met the minimum recommendations for UK physical activity levels [2]. The problem of inactivity presents a considerable concern to the general public; however, these effects are even more serious for those who have suffered an amputation. Multiple studies indicate that the likelihood of participating in physical activities, following an amputation, decrease if they did not participate in activities prior to the amputation [3]. Current physical activity recommendations for an adult in the UK suggest that at least 150 min of moderate intensity exercise should be carried out, on a weekly basis. Typically recommended activities include brisk walking and cycling [2]. The limb-absent population is no exception to these guidelines. Those who do return to leisure or sports activities tend to opt for less strenuous activities, such as swimming and fishing, where either a prosthesis is not needed or is not functionally required for participation. A review of 12 independent studies indicates that 68% of the amputee population is generally inactive [3]. Following an upper-limb amputation, leisure pursuits become more sedentary and indoor in nature [4]. Moreover, arms and hands are required for most daily activities, as well as being essential for communication and showing affection [5,6]. Loss of an upper limb therefore results in serious restrictions to function, sensation and appearance [7].

Upper-limb amputations occur less frequently than lower-limb amputations and typically result from traumatic injury in healthy, young adult, male individuals [8,9]. Studies suggest that the average age of upper-limb amputees, at amputation, varies between 20 and 36 years [10,11,12,13]. Estimates from 2005 suggest that the prevalence of major upper-limb loss in the USA was 41,000 [14] and 280 in the UK [15]. Following a major upper-limb amputation, multidisciplinary rehabilitation is required [16].

In rehabilitation through sports, cycling is considered one of the best forms of exercise for amputees, since it is a muscle-strengthening activity and involves relatively low joint loadings [17]. There are various opportunities for lower-limb amputees to participate in cycling, including rigid leg sport-prostheses and hand-cycles; however, there are only limited options for upper-limb amputees. In order to allow for these people to engage in an active lifestyle, there is a need for a low-cost upper-limb sports-prosthetic device. The NHS service specification for people of all ages with limb loss includes a provision for recreational prosthetic appliances and components, in order to meet the clinical needs and rehabilitation goals of an individual [18]. If the individual previously participated in cycling, a cycling terminal device could allow these activities to be integrated into the rehabilitation process. This article describes the design and development of an upper-limb cycling prosthesis for rehabilitation and recreational purposes.

## 2. Materials and Methods

### 2.1. Current Designs and Limitations

Participation and interest in Para-Sports has increased drastically in recent years. The London Paralympic Games in 2012 were televised in more countries than ever before and attracted the biggest ever audience [19]. These events have been valuable to increase public awareness of the capabilities of amputee athletes and the disabled population in general. Studies have noted that participation in sports and recreation is important for persons with limb deficiency for their reintegration into the community [20]. Opportunities to participate in leisure and competitive sports have improved through a growing public interest in physical fitness and accompanying sports organizations for the disabled [20]. Advances in prosthesis design, componentry and fabrication have also been an essential part of this development. The use of a functional prostheses is quite difficult for most proximal amputations, which are associated with higher rejection rates [12]. Only 37% of upper-limb amputees use their prosthesis regularly in the long term, with 19% being occasional users [4]. A third of people with a limb deficiency only require a cosmetic prosthesis, for basic functions in daily life [21,22]. These are considered ‘passive function prostheses’, as they are used for steadying and supporting [23]. However, some upper-limb amputees are likely to favour a so-called activity limb, which is dedicated for sports-related activities. Others may prefer an artificial limb with different terminal devices that can be interchanged, depending on their lifestyle, occupation and leisure activities [21]. In addition to standard aspects of prosthesis evaluation (comfort, fit, weight, hygiene, etc.), the demands of the desired activity and the environment in which the sport is to be played need to be considered. Sport-specific design efforts have produced a range of upper-limb activity limbs, suitable for sports such as basketball and skiing [24].

When controlling bicycle handlebars, there are three key considerations: control, flexibility and release. The human hand offers very good control, many degrees of flexibility and release by simply letting go. When wearing a prosthesis, this is not possible. Current designs approach this problem with varying degrees of success, often sacrificing one factor for another. Some designs approach this issue by offering a rudimentary design, with a rigid connection to the handlebar. Professional devices (appropriate for the Paralympic level) consist of a customised socket, to match the residual limb, so that the rider can easily detach from the handlebar. However, amateur and semi-professional variants of this design involve the rider being attached, or ‘strapped on’, to the handlebar at the beginning of the race. In the event of a crash or accident, these designs rely on mechanical failure to release the rider. Failure of a prosthesis during either recreational use or competition can result in physical injury and severe psychological repercussions [20].

To combat this, some designs offer flexible connections, such as a socket and pin which replicates wrist movements, whilst maintaining a fixed connection to the handlebar. In these cases, transradial amputees tend to wear a loose-fitting flexible socket, from which the residuum can easily be removed in the event of an accident. Although improving prosthetic flexibility, socket and pin connectors are still limited by a mono-directional release mechanism [24]. The rider is required to push their arm up and forwards to release. In the event of a crash, it is unlikely that this will always be possible. Facing a crash with an arm still attached to the bicycle, the amputee may lose the opportunity to break their fall. The arm could even release from the socket, leaving the rider to land on their residual limb.

Alternative designs use a ball-connector that sits in a socket, attached to the handlebar. This solution offers greater control and even more flexibility. The ball is typically secured using a simple friction fit, offering greater movement to adjust riding positions and an easy release mechanism. However, this design is often perceived to offer too much flexibility for some users, since freedom of movement results in limited steering control [25]. Moreover, commercially available products such as Mert’s Hands start at $2000 [26]. Alternatively, $10,000 are required to purchase a transradial upper-extremity prosthesis with a functional ‘split hook’ device for below-the-elbow amputees [27]. The lack of affordable and commercial cycling prostheses presented an opportunity to develop an upper-limb sports prosthesis, which could viably outperform what the market currently offers.

### 2.2. Novel Design Concept

To devise a novel concept for an effective release mechanism, fundamental knowledge in regard to the correct and recommended behaviour during a crash was required. Some crashes are simply too violent and sudden to react to, especially involving collisions with another vehicle. Nonetheless, experts suggest that crashing is an important skill for cycling and that it must be practiced in order to be performed safely [28]. In 2014, 16% of reported cycling accidents resulted from a loss of control. Moreover, driver or rider errors contributed to 73% of cases [29]. Based on these findings, the team decided that two safety mechanisms would be required; one for instances in which the rider has partial control and can initiate the release themselves and another which can release automatically in situations too sudden for the rider to react to. When a rider loses control, they often have only a few moments to react. The correct behaviour in these situations can significantly reduce the risk of serious injury. The rider’s hands need to get “off the bars and out” from the handlebars, to separate from the bicycle [28]. Hands and forearm should make first contact with the ground; however, it is imperative that the arm is not held outstretched, which can result in a broken collarbone [30]. Instead, the arm must be free to protect head and body during impact [27]. Based on these recommendations, the concept of the upper-limb prosthetic was designed to enable the rider to free their arm and initiate a rolling action, in the event of a crash.

**Figure 1:**
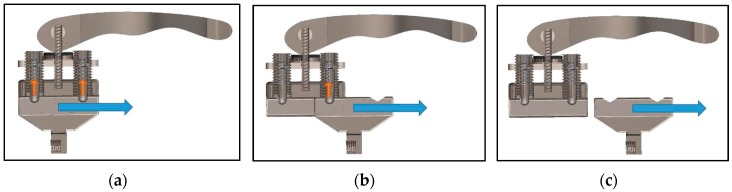
Sliding release mechanism: (**a**) Locked; (**b**) Intermediate Release; (**c**) Complete Release.

The specification and requirements for an ‘ideal’ prosthesis were developed in consultation with prosthetists. Standardised and commercially available components were used to reduce the overall manufacturing costs, enable simple servicing and ensure that the new design could be integrated with existing terminal devices. The proposed emergency release mechanism allows the prosthetic hand to release by sliding parallel to the handlebars, in either direction, depending on which way the rider is going to fall. A sliding bar serves as a simulated hand, which is able to recreate the desired motion during a crash. Spring plungers were included in the design to prevent the slider from releasing accidentally. Unless sufficient force is applied to overcome the plungers, the bar is rigidly connected to the handlebar. The force required by the rider to initiate the sliding motion will most likely vary from rider to rider. Two adjustable spring plungers were implemented to provide a wider range of operational forces. The second plunger acts as a redundancy, in case one fails unexpectedly. The release mechanism, shown in Figure 1, demonstrates the transitions from an initially locked position to sliding out of the housing.

If enough sideways force is applied by the rider, the hand begins to slide out by overcoming the force required to push the internal springs of the plungers up. The second plunger is overcome twice, as shown in Figure 1b, preventing accidental release. If enough force has been applied to overcome the second spring plunger, the slider releases from the middle-block entirely, as shown in Figure 1c.

The sliding release mechanism allows the user to ‘let go’ of the handlebar and protect head and body during impact. The limitation of this design is that the mechanism will not be effective during a head-on collision. In such crash scenarios, the rider cannot be realistically expected to apply a side-ways motion to release.

A secondary release mechanism was designed, intended to free the rider from the handlebar in accidents too sudden and/or violent to which to react. A so-called pylon provides a structural connection between the residual limb liner and end-effector (i.e., slider hand). The pylon thereby simulates the amputee’s forearm. A bicycle seat clamp was used to secure two halves of the pylon by radial friction forces, which require an axial force to release the tubes from the clamp. This clamp connection can be reassembled easily following an incident and does not rely on mechanical failure to release.

### 2.3. Experimental Measurement Setup

An important consideration for the mechanism was the selection of appropriately sized spring plungers, which in turn provides the range of forces appropriate for most users to initiate release. To determine this, potential rider force data needed to be collected. An experiment was designed and executed, which involved a digital force meter, an immobilised vice and an able-bodied volunteer. The volunteer was a 23-year-old, healthy, male individual. The age and sex of our volunteers match the demographic of individuals most frequently affected by upper-limb amputations.

**Figure 2:**
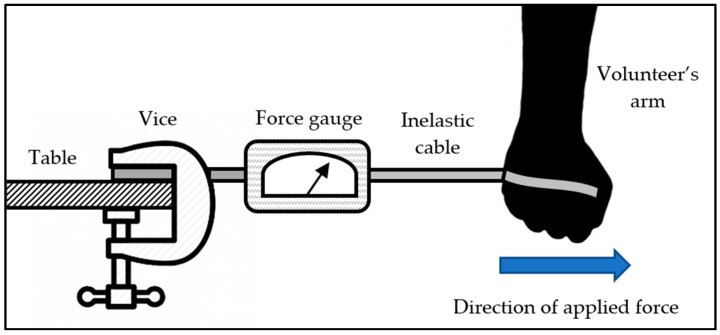
Experimental setup to measure release forces.

A vice was secured to a table and attached to a digital force gauge (CFG+, Mecmesin, West Sussex, UK). The other end of the force gauge was attached to an inelastic cable, which the volunteer tied around the palm of their hand. The experimental setup is shown in Figure 2. The force gauge was set to record maximum force. The volunteer was asked to initiate a sudden lateral motion, away from the force gauge. The experiment was repeated 3 times. It was determined that a mean force of approximately 200 N could be applied by our volunteer.

To determine the forces required to initiate release of the secondary mechanism, the experiment in Figure 2 was repeated. The volunteer’s arm was replaced with the two pylon halves, secured with the bicycle seat clamp. One end of the pylon was connected to the inelastic cable, while the volunteer pulled on the other end to simulate an axial force. The force gauge was set to record maximum force to determine how much force is required to release the pylon halves. It is difficult to estimate what forces will be experienced during a sudden accident; however, the volunteer was unable to release the pylon halves beyond a maximum force of 500 N. The radial friction forces of the bicycle seat clamp were increased to approximately 700 N. This prohibits the user from initiating the secondary release accidentally. External forces, experienced during an accident, are required for the pylon halves to separate.

The specification of a prosthesis is often tailored to the individual. The volunteer had agreed to participate in further experimentation to evaluate the prosthesis. The spring plungers used for the design prototype were selected to match the requirements from the results of the experiment described above. In this case, the prosthesis was intended for a male subject in his early 20 s. This procedure simulates the customisation process that is likely to occur when a prosthetist modifies a prosthesis for their patient. It is worth noting that the able-bodied individual has intact musculature and a potentially longer moment arm than an amputee. However, by referring to the maximum force applied by the able-bodied volunteer, we believe that the maximum force required for an amputee subject (in the same demographic) is likely to lie below this value. The technique used for selecting spring plungers is therefore suitable to account for most inter-person variability.

**Figure 3:**
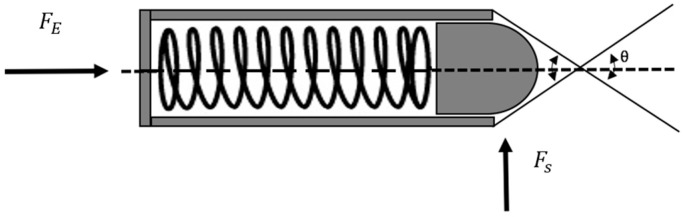
Diagram of the spring plunger forces.

To determine the force required to push the spring plungers to release the slider, Equation (1) was used (in reference to Figure 3).

(1) $$F_{S}=\frac{F_{E}}{\tan\left(\frac{\theta }{2}\right)}$$

The angle of the V-shaped groove determines the sliding force required to release the plunger. $F_{S}$ is the force applied by the rider, which from the previous test was determined to be around 200 N. $F_{E}$ is the compression force of the spring plungers. This information was provided by the supplier of the spring plungers (Norelem, Birmingham, UK).

The vector sliding forces sum to produce a total sliding force. Therefore, the force required from each spring plunger must be halved. The optimal angle of grooves achievable in manufacturing was determined to equal 60°, as a result of using a 30° cutting tool. This gave a target $F_{E}$ of 57 N, shown in Equation (2).

(2) $$100=\frac{F_{E}}{\tan\left(\frac{60}{2}\right)}$$

**Figure 4:**
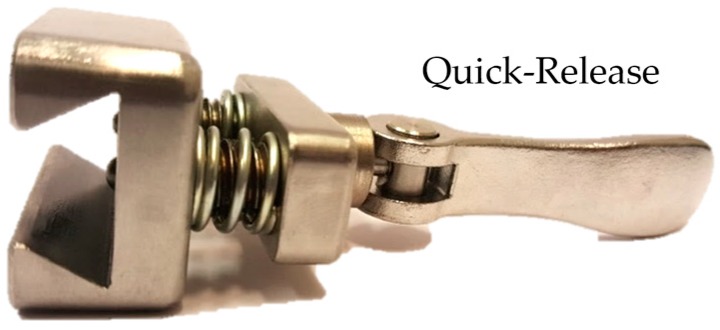
Spring plungers with quick-release.

M12 spring plungers were used, giving a range of: $F_{E}\left(\mathrm{Min}.\right)=19\;N\;\mathrm{and}\;F_{E}\left(\mathrm{Max}.\right)=74\;N$. The total force required to initiate release can therefore be adjusted between the calculated range of: 141.8–256.3 N. A hex key is required to adjust the tension on the spring plungers. This range is likely to be sufficient for the mechanism to work effectively with male young adults. A wide selection of spring plungers can be used to achieve various ranges of forces, for different user demographics. Factors influencing the maximum force an amputee can exert may be affected by activity levels, body types and age. Moreover, the extent of the transradial amputation, i.e., length of the residual limb past the elbow joint, is likely to play a key factor. To aid detachment of the arm when stationary, a quick-release mechanism was included, as shown in Figure 4. This allows the user to pull and twist a lever to retract the spring plungers.

**Figure 5:**
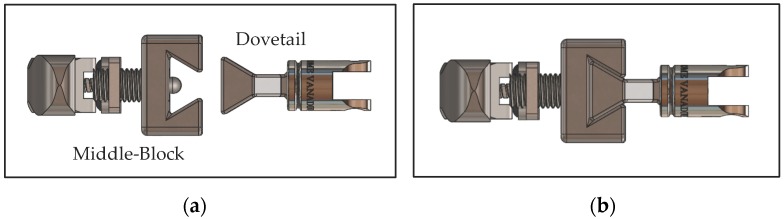
Dovetail concept: (**a**) Detached; (**b**) Attached.

Twisting the lever allows the user to adjust the protruding distance of the spring plungers, which results in a substantial reduction in force required to release the slider. To ensure that the slider was properly constrained when attached, the design utilises a dovetail concept between the slider and middle-block, demonstrated in Figure 5.

A key advantage of the dovetail mechanism is that it provides a secure connection between the slider and the middle-block, which can only release by applying a lateral force. A summary of the forces required to release the two emergency mechanisms is shown in Table 1.

### 2.4. Final Design

**Figure 6:**
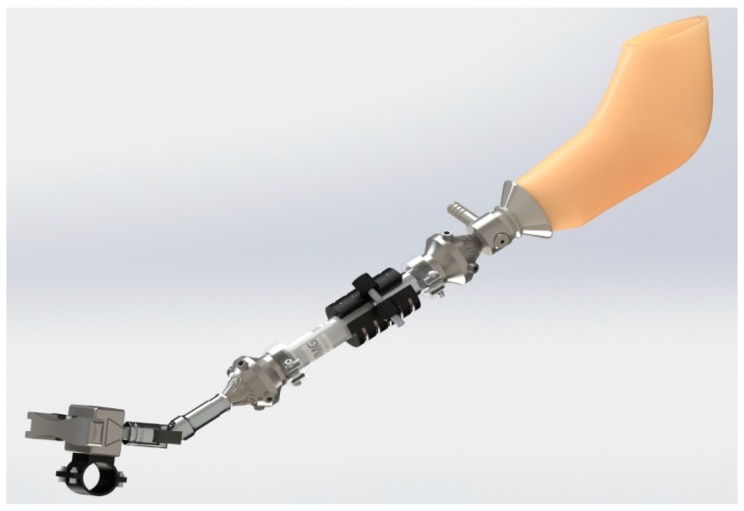
Final prosthesis design (assembled view).

**Figure 7:**
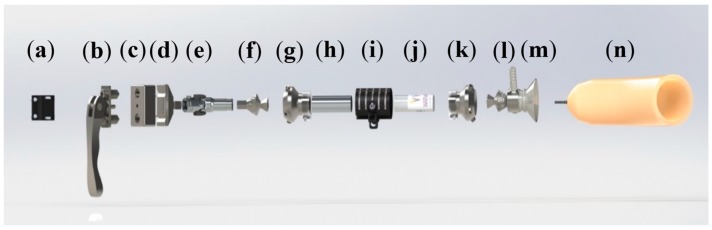
Final prosthetic design (exploded view): (**a**) Handlebar Connection; (**b**) Spring plunger release system; (**c**) Middle-block; (**d**) Slider; (**e**) Universal joint (with custom restrictor); (**f**) Male tube clamp adapter; (**g**) Female tube clamp adapter joint; (**h**) Lower-middle pylon; (**i**) Clamp release; (**j**) Upper-middle pylon; (**k**) Female tube clamp adapter joint; (**l**) Male tube clamp adapter; (**m**) Plastic cast; (**n**) *Iceross* Sport Liner.

The final prosthetic assembly is shown in Figure 6. Starting at the handlebar attachment, there is a slider block with a quick-release mechanism and spring plungers. The rest of the design consists of a restricted universal joint, male and female pyramid adapters, secondary clamp release, and cast attachment. An exploded view is shown in Figure 7.

The design is intended to compliment commercially available products, such as the *Iceross* Sport Liner. These liners are fitted with a locking mechanism, which involves a pin connecting to a plastic cast adapter, to form a secure and comfortable connection. Components (a)–(c) in Figure 7 will be attached to the bicycle handlebar, while components (d)–(n) will be attached to the rider.

Table 2 shows a breakdown of the manufacturing details relating to the components in Figure 7. Most parts were manufactured ‘in-house’ by workshop technicians at the School of Engineering.

**Figure 8:**
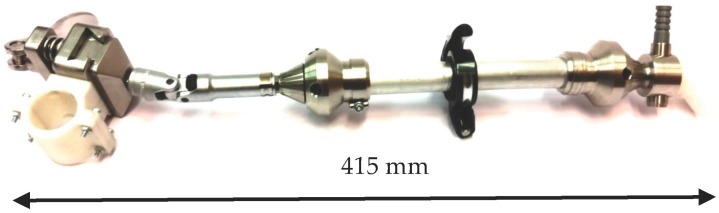
Manufactured upper-limb cycling prosthesis.

Pyramid connectors are standardised components used in a variety of prosthetic designs. These parts are very expensive and were therefore manufactured ‘in-house’ to further reduce costs. The estimated cost for materials and technician time for the device prototype is £500. Economies of scale and improved manufacturing processes can further reduce the production costs associated with this design concept. The manufactured prototype (shown in Figure 8) weighs 800 g and measures 415 mm.

## 3. Results

**Figure 9:**
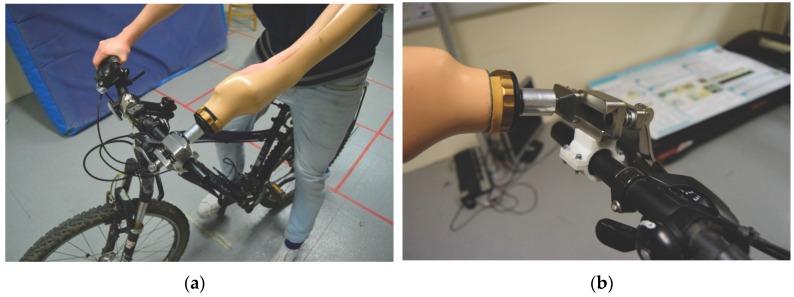
Practical experimentation: (**a**) Volunteer on bicycle; (**b**) Prosthetic hand release test.

The manufactured upper-limb cycling prosthesis was tested in a controlled lab environment to demonstrate the efficacy of the device during normal operation and to determine the reliability of the safety release mechanism, in the event of an emergency. The handlebar bracket fits all standard 7/8 inch (22 mm) diameter bars. A custom arm cast, shown in Figure 9, was used to allow the able-bodied volunteer to simulate a prosthetic hand.

The quick-release could be applied with one hand to attach and detach the slider ‘hand’ during mounting and dismounting. The test-rider was successfully able to stabilise on the bicycle. The spring plungers were perceived to provide ample resistance and, when locked, allowed the rider to have full control of the handlebars to steer and complete basic turning manoeuvres. The volunteer was then stabilised by helpers and asked to release the slider from the handlebars by applying a sideways force. The test-rider was able to free his arm quickly, freeing it to protect head and body during a sideways fall. This matches the calculations of the upper resistance limit of about 200 N. If the user desired a lower resistance limit, the spring plungers could be simply adjusted by twisting the quick-release (to further retract the spring plunger) or using a hex key to adjust the tension on the spring plungers.

A new prosthesis is often evaluated by a prosthetist using a subject-reported experience. The assessment is intended to evaluate factors including: prosthesis suspension, socket comfort, socket fit, reliability, functionality, usefulness, weight, appearance and shape [25]. The factors are rated on a scale of: not at all, a little, somewhat, quite a lot, a lot; relating to how restricting they are in participating in activities. Since the experiment was conducted with an able-bodied subject, the factors of reliability, functionality, usefulness, appearance/shape were evaluated, as shown in Table 3.

The results from the subject-reported experience are encouraging. Usefulness, reliability and functionality factors were perceived as ‘not at all’ and ‘a little’ restricting to participate in activities. The appearance/shape is acceptable with its functional design; however, the weight is perceived as too heavy and restricting.

## 4. Discussion

For prosthetic devices that are intended for recreation and sports activities, cosmesis is of lower priority than functionality. However, the additional mass of the design poses a potential concern. Research indicates that the metabolic cost during walking is unchanged up to a mass of 1.3 kg using a transfemoral prosthesis [31]. There is little known about the metabolic effects of prosthesis mass on higher level activities, such as cycling. Based on the results from the subject-reported experience, the foremost issue seems to be weight. The current prototype weighs 800 grams and would require an amputee to exert quite a lot of force through their residual limb. A major development for the arm design could involve an experiment to precisely quantify the forces an amputee would be able to exert during the general use of the prosthesis. This data could then be used to precisely allocate a number of angular turns of the quick-release for specific user demographics. Additionally, some of the parts could be machined out of lighter materials, such as aluminium or titanium alloy, instead of steel. Additive manufacturing techniques could even enable various parts to be 3D-printed. Extensive testing will be required to simulate and experimentally determine the mechanical properties of these components.

The qualitative evaluation is very subjective, due to only having one volunteer. This information can therefore only provide a rough indication of how an amputee user may assess the prosthesis. However, these impressions may serve as a valuable reference for future studies, where we hope to test and evaluate the prosthesis with 2–5 upper-limb amputee volunteers.

The length of the current design can be altered by cutting longer or shorter aluminium tubes to better suit the rider’s forearm length. A more advanced solution could involve a design which adapts its length through a gear and pinion mechanism to create a ‘one-size-fits-all’ design. Children may require a new prosthesis once every three or four years until age 21 [27]. This mechanism can be used to adapt the prosthesis pylon length to match the development of the user.

The novel mechanism proposed in this article will likely require users to attend training sessions to learn and practice applying the mechanism safely, in a controlled environment. However, it is not uncommon that persons using a new prosthesis for sports require extensive training to practice the safe use, maintenance and adjustment of the device [20].

Due to the rigid design, the rider may experience shocks when riding over uneven surfaces. The significant biomechanical forces and repetitive nature of turning motions is likely to strain the interface with the residual limb. Interface materials such as silicone liners, gel liners, or hypobaric socks could be used to provide additional padding to help absorb and disperse pressure and shear forces. Moreover, the middle pylon could be replaced with a full suspension system to overcome this issue. When cycling on rough terrain, shocks and impacts exerted through the rider’s arm could be absorbed and dissipated through a spring-based damping system. However, further work is required to determine the likely range of forces experienced during an average journey, e.g., riding on a dirt road or bike path.

## 5. Conclusions

A comprehensive assessment of the current drawbacks of transradial prosthetics in the field of cycling was presented. Based on these findings, a novel upper-limb cycling prosthesis was designed and developed. The design includes two release mechanisms, including a sliding mechanism for falls and minor collisions and a clamping mechanism for head-on collisions. The former requires the rider to exert about 200 N of force, while the latter releases if over 700 N are applied. The required forces can be customised to match the preferences of the rider. The design prototype was manufactured and tested by a volunteer test-subject to demonstrate that the design offers functionality and stability during normal operation and allows the rider to reliably activate the safety release mechanism, in a simulated emergency scenario. The total cost of the prototype is estimated at £500. The development of the device proposed in this article could allow people with upper-limb amputations to participate in cycling for rehabilitation or recreational purposes.

## Figures and Tables

**Table 1:** Release mechanism summary.

Crash Scenario	Release Mechanism	Required Force
Falling Left/Right	Slider	200 N
Head-on Collision	Clamp	700 N

**Table 2:** Manufacturing details for upper-limb cycling prosthesis.

Part Name	Description	Material	Manufacturing Process	Qty
Slider	End-effector, which slides laterally if force is applied	Steel	Milled from solid steel block	1
Middle-block	Housing secures slider/connects to handlebar	Steel	Milled from solid steel block	1
Spring plungers	Provide variable locking forces to secure slider	Steel	Manufactured by Norelem	2
Quick-release	Allows for easier release and adjustment of slider	Steel	Manufactured by Thorn Cycles	1
Handlebar brackets	Connect slider housing to handlebar	ABS	Fused deposition modelling	1
Universal joint	Simulates wrist and allows limited movement	CrV	Manufactured by Faithfull	1
Restrictor	Limits excessive universal joint movement	Neoprene	Manufactured by RS Pro	1
Pyramid Connector	Male/female joint adapters	Steel	Machined using manual lathe and CNC mill	4
Pylon	Rod that provides structural support	Al	Metal turned	2
Seat Clamp	Secures two pylon halves	Carbon fibre	Manufactured by IMUST	1
Plastic Cast	Interface to *Iceross* Liner	ABS	Fused deposition modelling	1
*Iceross* Liner	Protects residual limb and connects to plastic cast	Silicone	Manufactured by Ossur	1

**Table 3:** Qualitative evaluation—subject-reported experience.

Evaluation Criteria	Scale	Reasoning
Reliability	A little	Releases well, but will require training to apply quickly
Functionality	A little	Functions well, but needs to be tested in real-life scenario
Usefulness	Not at all	Very useful to increase participating in activities
Weight	Quite a lot	Heavier than expected
Appearance/Shape	Somewhat	Design is very functional, but appropriate for activities
